# Monitoring of the National Oil and Wheat Flour Fortification Program in Cameroon Using a Program Impact Pathway Approach

**DOI:** 10.1093/cdn/nzz076

**Published:** 2019-06-20

**Authors:** Henry E Mark, Jules G Assiene, Hanqi Luo, Martin Nankap, Alex Ndjebayi, Ismael Ngnie-Teta, Ann Tarini, Amrita Pattar, David W Killilea, Kenneth H Brown, Reina Engle-Stone

**Affiliations:** 1 Department of Nutrition, University of California, Davis, Davis, CA, USA; 2 Helen Keller International, Yaoundé, Cameroon; 3 Independent Consultant, Quebec, Canada; 4 Children's Hospital Oakland Research Institute, Oakland, CA, USA

**Keywords:** fortification, micronutrient, program impact pathway, implementation science, monitoring

## Abstract

**Background:**

Since 2011 Cameroon has mandated the fortification of refined vegetable oil with vitamin A and wheat flour with iron, zinc, folic acid, and vitamin B-12. In 2012, measured fortification levels for flour, and particularly oil, were below target.

**Objectives:**

We assessed Cameroon's food fortification program using a program impact pathway (PIP) to identify barriers to optimal performance.

**Methods:**

We developed a PIP through literature review and key informant interviews. We conducted interviews at domestic factories for refined vegetable oil (*n* = 9) and wheat flour (*n* = 10). In 12 sentinel sites distributed nationally, we assessed availability and storage conditions of fortified foods in markets and frequency of consumption of fortified foods among women and children (*n* = 613 households). Food samples were collected from factories, markets, and households for measurement of micronutrient content.

**Results:**

Two-thirds of factories presented quality certificates for recent premix purchases. All factories had in-house capacity for micronutrient analysis, but most used qualitative methods. Industries cited premix import taxes and access to external laboratories as constraints. Mean vitamin A levels were 141% (95% CI: 116%, 167%), 75% (95% CI: 62%, 89%), and 75% (95% CI: 60%, 90%) of target in individual samples from factories, markets, and households, respectively. Most industry flour samples appeared to be fortified, but micronutrient levels were low. Among composite flour samples from markets and households, the mean iron and zinc content was 25 mg/kg and 43 mg/kg, respectively, ∼45% of target levels; folic acid (36%) and vitamin B-12 (29%) levels were also low. In the previous week, the majority of respondents had consumed “fortifiable” oil (63% women and 52% children) and wheat flour (82% women and 86% children).

**Conclusions:**

In Cameroon, oil fortification program performance appears to have improved since 2012, but fortification levels remain below target, particularly for wheat flour. Consistent regulatory monitoring and program support, possibly through premix procurement and micronutrient analysis, are needed.

## Introduction

Different forms of malnutrition, including micronutrient deficiencies, contribute significantly to the burden of mortality and morbidity among young children and women of reproductive age (1). Staple food fortification is 1 strategy to reduce micronutrient deficiencies (2–4) in a cost-effective manner (5–7). Large-scale fortification programs have been implemented throughout many low- and middle-income countries in Africa, Asia, and South America (8, 9). Such programs are often advocated as “sustainable,” with the rationale that once the program is

launched, the effort to maintain the process is minimal, and the recurring costs for micronutrient premix procurement and industry compliance monitoring are absorbed by the consumer or the government. However, despite the efforts invested in initiating fortification programs globally, ensuring program compliance remains a challenge (8, 10). There is a growing appreciation of the need to understand barriers to implementation and to support monitoring systems (11) to ensure that these programs deliver meaningful and continuing impact.

A program impact pathway (PIP) analysis is a tool that draws on theory of change to identify key inputs, processes, and outputs in the causal chain through which a program is hypothesized to achieve its intended impact (12). By monitoring key points throughout the PIP, program managers can highlight bottlenecks in program implementation to guide management decisions and instigate corrective actions where needed. The PIP approach has been applied successfully to various nutrition intervention programs, including homestead food production (13), school-based healthy lifestyles programs (14), micronutrient powder distribution (15), and programs to improve infant and young child feeding and sanitation and hygiene (16–18). In addition to guiding program management decisions, this information is critical for determining the timing of impact evaluations (19, 20).

Micronutrient deficiencies have been a long-standing public health challenge in Cameroon (21–24). Informed by estimates of the reach of potentially fortifiable staple foods (21), as well as feasibility and cost estimates, the government of Cameroon mandated fortification of all refined vegetable oil and wheat flour, with distribution of fortified products commencing in August 2011. In 2012, an initial impact evaluation in the 2 largest cities in Cameroon, Yaoundé and Douala, showed no impact on vitamin A status in women or children (25), possibly because only 44% of household oil samples were fortified with vitamin A. In contrast, wheat flour samples collected from markets were fortified at ∼75% of target levels, and the status of iron, zinc, folate, and vitamin B-12 (all nutrients added to wheat flour) was greater among both women and children compared with prefortification values (26).

Following these observations, we aimed to provide insights into the challenges and opportunities for sustaining large-scale food fortification programs using the case of mandatory fortification of vegetable oil and wheat flour in Cameroon. Guided by a PIP, the results of data collection at industries, as well as markets and households within sentinel sites, were used to identify specific points along the expected causal pathway for programmatic attention to ensure that the program achieves the intended impact on micronutrient status and health.

## Methods

### Development of a PIP

**FIGURE 1:**
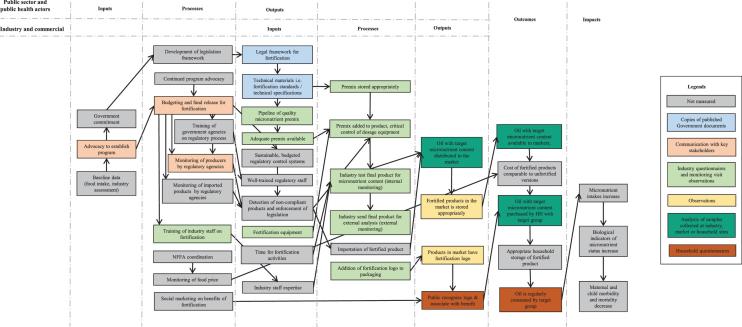
PIP and relevant data sources for assessing a large-scale edible oil fortification program. Box colors indicate data sources used to evaluate the PIP for Cameroon. The arrows represent the linkages between elements of the PIP and are intended to show the major associations rather than being exhaustive. Although the format is linear, we acknowledge that many of the relations within the PIP are cyclical and that many of the feedback loops in the program occur simultaneously. HH, household; NFFA, National Food Fortification Alliance; PIP, program impact pathway.

We developed a PIP through reviewing the literature and interviewing key informants to conceptualize the critical inputs, processes, outcomes, and desired impact of the national food fortification program. **Figure 1** shows the PIP for refined oil fortification, indicating the data sources used to assess each aspect of the pathway. The PIP for the wheat flour program is similar, but it omits the importation of wheat flour because the vast majority (>∼95%) of wheat flour is milled domestically in Cameroon. The PIP distinguishes the roles and perspectives of the public sector and public health actors from those of the industry and commercial organizations, in that the outputs of processes managed by the former (e.g., legislation and technical standards and a permanent regulatory system) serve as inputs to the industry processes that lead directly to availability of the fortified product on the market and thus the expected program impacts. In this analysis, we focus primarily on the industry perspective. The PIP was then used to guide the creation of data collection tools. For each box of the PIP, a priori criteria were set to determine the functioning of the specific component, categorized as working well, needs minor improvement, needs major improvement, or not working. Full details of the definitions, performance criteria, and indicators are available in the supplementary material (**Supplemental Tables 1** and **2**). Modifications made to the PIP following data collection are noted.

### Selection of industry sites, markets, and households

All industrial-scale oil refiners and wheat flour millers in Cameroon that were operational at the time of data collection were eligible for inclusion in the study. For the market and household data collection, a total of 12 clusters were purposively selected from the 90 clusters used in the 2009 national nutrition survey (21). Criteria for the selection of clusters included *1*) geographic representation—1 site in each of 10 administrative regions and 1 in each of the major cities of Yaoundé and Douala; *2*) representation of both urban and rural areas in the “North” and “South” macro-regions of the 2009 survey; *3*) availability of refined oil and wheat flour during the 2009 survey (eligible if wheat flour and oil were consumed by ≥20% of women and/or children in the past week); *4*) accessibility by road in all seasons; and *5*) security. Data were collected between June and August 2016.

### Monitoring visit procedures

#### Industry sites

Each industry was visited by a study team composed of individuals from the government of Cameroon (Ministry of Industry and Ministry of Health), Helen Keller International, and the University of California, Davis, on a date arranged with the factory. During the monitoring visit, questionnaires were administered to the industry representative by a single interviewer, and observations were made by the monitoring team. Information was collected on quality assurance and control of the fortification process, including the availability of both qualitative testing methods (tests that indicate the presence of the micronutrient of interest in a sample, such as the iron spot test) and quantitative testing methods (tests that show the quantity of the micronutrient of interest in a sample, such as the iCheck instrument for analysis of vitamin A in fortified oil).

At both oil factories and wheat millers, composite samples of final products were requested at the time of the visit for each lot number of each brand that was available at the time of the visit. The number of lots included in a composite sample depended on availability at the time of the visit and ranged from 1 to 3 for oil samples and 1 to 2 for flour samples. Up to 5 samples (depending on availability, each from different packages) from each available production lot for each brand were requested and then combined to prepare a composite sample for each brand. Sample containers were wrapped in aluminum foil to prevent light exposure. All industry food samples were stored in a cooler with ice packs after collection until transfer to a freezer at the end of each day. Samples were stored at –20°C until analysis.

#### Markets

Samples of refined vegetable oil (∼10 mL) and wheat flour (∼50 g) were collected by enumerators from a convenience sample of markets and vendors in each cluster (primarily open-air market stalls and small retailers but also including supermarkets and mobile vendors). After completing the household data collection, nearby vendors were visited until the target sample size (∼50 samples per cluster, as described later) was achieved. All oil samples and a 20% subset of wheat flour samples intended for B vitamin analysis (i.e., ∼10 flour samples per cluster for market and household samples combined) were wrapped in aluminum foil, stored in a cooler with ice packs until transfer to a freezer at the end of each day, and stored at –20°C until analysis. The remaining wheat flour samples for mineral analyses were stored at room temperature.

#### Households

The household survey was conducted to collect information on the downstream results within the PIP (Figure 1). Households within clusters were sampled using a random starting point determined by a modified random walk method: numbering houses from the center of the cluster to the edge in a prespecified random direction (expressed as degrees from North) and randomly selecting a starting point along this line. Subsequent households were identified by systematic selection of the next household to the right. Households were eligible to participate if at least 1 child aged 6 mo to 15 y and a woman of reproductive age (15–49 y), who was the child's primary caregiver, resided there. If multiple children within the selected age range were present in the household, 1 child was chosen at random to be the index child.

Trained interviewers conducted the household interviews with respondents. Written informed consent was obtained from the primary adult respondents for themselves and for the participating child prior to data collection. The protocol was approved by the National Ethics Committee of Cameroon (2016/05/717/L/CNERSH/SP) and the University of California, Davis, Institutional Review Board (#795701).

An FFQ, identical to that used in previous surveys in Cameroon (21, 26), was administered to caregivers to determine the coverage and frequency of consumption of refined vegetable oil and wheat flour for both themselves and the index child. First, respondents were asked on how many days during the past 7 d they had consumed each food item in different preparations (i.e., wheat flour in bread, pasta, and beignets); subsequently, respondents were asked how many times on the most recent day of consumption they had consumed each food item. The questionnaires also included information on household demographics, indicators of socioeconomic status, and food security, assessed using the Household Food Insecurity Access Scale.

Where available at the household, refined oil and/or wheat flour samples were requested by the interviewer (∼10 mL refined oil or ∼50 g wheat flour, following thorough mixing of the food). Samples were stored and transported as described for market samples, with all oil samples and a 20% subset of wheat flour samples included in the cold chain.

### Sample size

The target sample size for fortified food collection was 50 samples of each food vehicle per cluster from a combination of markets and households. This sample size was selected as sufficient to detect a proportion of food samples that were adequately fortified (i.e., meeting 80% of the national fortification standards) with ∼11% precision at the site level and ∼3.2% precision at the aggregate level. This sample size calculation applies to oil samples, which were individually analyzed, but not to wheat flour samples, for which brand- and cluster-specific composite samples were prepared.

### Food sample analysis

Food samples were shipped on dry ice to Davis, California, for micronutrient analysis. The vitamin A content of individual oil samples was measured using the iCheck Chroma 3 (BioAnalyt) according to manufacturer’s instructions. Measured values for the iCheck instrument control were within the appropriate range and varied 2.3% during the course of the analysis. A pooled fortified oil sample was measured at the beginning of each batch of samples and confirmed to be within 10% of the calibrated value before continuing the analysis of unknown samples. The variability of the vitamin A content of the pooled sample over 26 sample analysis runs was 5.2% within runs and 13.0% between runs.

For analysis of iron and zinc concentrations in wheat flour, composite samples for each combination of brand, cluster, and market or household were prepared in the laboratory by combining the individual samples of the same brand collected from either markets or households in that site. The number of individual samples in each composite sample ranged from 1 to 28 (**Supplemental Table 3**). Mineral content was measured by inductively coupled plasma optical emission spectrometry at the Children's Hospital Oakland Research Institute (26). For the analysis of folic acid and vitamin B-12 content of flour samples, we created cluster-level composite samples by combining individual market and household samples from a given cluster if they were included in the 20% subset retained in the cold chain. Vitamin content was measured by microbiological methods (AOAC International methods 992.05 and 960.45 for folic acid and methods 952.20 and 960.46 for vitamin B-12) at Covance Laboratories.

### Statistical analysis

Data were analyzed with Stata 14 IC (Stata). Each element of the PIP was assessed against prespecified criteria to categorize each element and facilitate the identification of problematic elements within the PIP. The criteria were developed through literature review, key informant interviews, and consensus among the research team members (Supplemental Tables 1 and 2). For industry-level data, we summarized information collected from wheat flour millers and oil producers separately. For market and household data, descriptive statistics were calculated by cluster and overall for oil and wheat flour. Overall SEs were estimated with survey procedures to account for the cluster design. This was also the case for the micronutrient content of oil samples but not wheat flour samples, which were analyzed as brand–cluster composites.

We summarized micronutrient content of oil and flour samples by cluster and brand and also expressed these values as percentages of the values included in the fortification norms of Cameroon (**Table 1**) (27, 28). Note that the norms specify the total amount of *added* micronutrient, but the values measured in wheat flour samples represent *total* iron and zinc. Thus, we present total iron and zinc content as well as estimates of added iron and zinc, which were calculated by subtracting an estimate of intrinsic iron and zinc from each measurement. Intrinsic iron and zinc content was assumed to be 12.5 mg/kg iron and 8.5 mg/kg zinc, the mean iron and zinc concentrations in 9 unfortified samples collected during a previous study (26).

**TABLE 1:** Mandatory micronutrient fortification standards for wheat flour and refined vegetable oil in Cameroon

Micronutrient, chemical form	Fortification levels in wheat flour^1^ (27)	Fortification levels in refined vegetable oil (28)
Iron, as ferrous fumarate, mg/kg	60 ± 6	—
Folic acid, mg/kg	5.0 ± 0.5	—
Zinc, as zinc gluconate, mg/kg	95 ± 9.5	—
Vitamin B-12, cyanocobalamin, mg/kg	0.04 ± 0.004	—
Vitamin A, retinyl palmitate, mg/kg	—	12.0 (9.9–15.0)

^1^Standards are expressed as the target ± 10%.

Regression models, accounting for clustering in estimation of SEs, were used to compare the mean micronutrient content of samples collected from markets and households. Where no significant difference was observed between market and household samples, the data were pooled for comparison with composite samples from factories.

Reach of fortifiable oil and wheat flour was defined as consumption by the target population group at least once in the previous week. Frequency of consumption of wheat flour-containing products was calculated by multiplying the number of times per week a wheat flour-containing food was consumed by the number of times the food was consumed on the last day of consumption. Total frequency of wheat flour intake was then calculated by summing the weekly frequency of intake of all wheat flour-containing products excluding pasta, which is not included in the fortification program. Frequency of fortifiable oil intake was calculated in a similar manner, excluding products produced with red palm oil and groundnut oil, which are not industrially refined in Cameroon and thus not considered “fortifiable.” Regression models, accounting for clustering, were used to assess differences in reach and frequency of consumption of wheat flour and oil among clusters.

## Results

### Summary of data collection

A total of 12 wheat flour millers and 12 oil producers were identified for the study. Data collection was completed for 10 wheat flour millers and 9 oil producers, with the remaining factories having ceased production or not commenced operations. Combined, the 10 flour millers produced a total of 35 brands of wheat flour, and the 9 oil factories produced 15 brands of refined vegetable oil (**Table 2**). At the market level, refined oil samples were collected from a total of 256 vendors (**Table 3**), of which 53% were market stalls, 34% were small retailers, and the remainder were supermarkets, mobile vendors, and other points of sale. Wheat flour samples were collected from 206 vendors, of which 61% were markets stalls, 27% were small retailers, and the remainder were supermarkets and other points of sale. A total of 613 household interviews were completed in the 12 clusters. On average, participating women were 31 y old, and the index children were 6 y old (ranging from ∼6 mo to 15 y).

**TABLE 2:** Characteristics of monitored industry sites

	Flour millers (*n* = 10)	Oil refiners (*n* = 9)
Production		
Factories have automated system for adding and mixing fortificants	8/10	5/9
Median brands produced per factory (IQR, absolute range) [*n*]^1^	3.5 (3, 1–6) [10]	1 (1, 1–5) [9]
Median annual factory production in 2015 tons (IQR) [*n*]	28,755 (44,221) [7]	4112 (2665) [8]
Premix usage		
Median amount of premix used (kg) in 2015 (IQR) [*n*]	15,042 (20,654) [8]	162 (135) [9]
Median cost in USD per kilogram of premix (IQR) [*n*]^2^	8.58 (5.24) [6]	62.0 (15.8) [7]
Certificates of premix quality available	7/10	6/9
Instructions for the receiving of premix are available	6/10	7/9
Instructions for the storage of premix are available	5/10	7/9
Appropriate premix storage	6/10	6/9
Report of premix stock out in past 12 mo	0/10	0/9
Median premix used as percentage of premix required to adequately fortify the total production reported (IQR) [*n*]	111% (43%) [5]	69% (38%) [8]
Quality assurance and control		
Written instructions on control of dosage equipment available	10/10	9/9
Records of dosage equipment calibration available	6/10	6/9
Records of flow rate monitoring available	7/10	6/9
Some form of in-house micronutrient test method available	10/10	9/9
Equipment and reagents for quantitative micronutrient analysis available	1/10	3/9
Equipment and reagents for semiquantitative micronutrient analysis available	0/10	2/9
Equipment and reagents for qualitative micronutrient analysis available	10/10	6/9
In the past 18 mo staff have been trained on quality control for fortification	2/10	1/9
Reported external laboratory analysis ever conducted on final product	5/10	3/9
Evidence of external analysis conducted on products within past 18 mo	3/10	2/9
Product storage and packaging		
Final product stored appropriately^3^	10/10	9/9
Factories that have the fortification logo on all brands they produce	10/10	9/9
Factories that have social marketing that focuses on the logo	4/10	9/9

^1^The number of factories from which data were available.

^2^USD equivalent on 30 June, 2015, cost excluding tax and freight. Premix procurement documents for 3 factories did not specify taxes paid.

^3^Products stored off the floor and out of direct sunlight.

**TABLE 3:** Storage conditions of refined oil and wheat flour samples in markets, by cluster^1^

Food vehicle	Cluster, region	Vendors, (individual samples)	Exposed to light, %	Exposed to air, %	Original package, %	Labeled as fortified, %
Wheat flour						
	Sabongari, Adamaoua	28 (28)	35.7	85.7	100	100
	Nkolkougda I, Central	10 (10)	100	100	80.0	80.0
	Bilongue II, Douala	24 (24)	100	100	100	100
	Bingomo, East	23 (25)	20.0	44	100	100
	Founaguedje, Far North	35 (38)	5.3	97.4	100	97.4
	Kombi III, Littoral	14 (25)	100	100	100	100
	Wouro Lawan, North	27 (35)	0.0	88.6	100	100
	Ntambeng II, Northwest	5 (21)	90.5	38.1	47.6	47.6
	Ngeme, Southwest	5 (14)	100	100	100	100
	Bachepang, West	16 (26)	100	100	100	100
	Djoungolo I, Yaoundé	19 (21)	95.2	95.2	90.5	95.2
	Total	206 (267)	58.1	86.1	94.4	94.4
Refined vegetable oil						
	Sabongari, Adamaoua	22 (35)	24.3	37.8	86.5	86.5
	Nkolkougda I, Central	16 (36)	50.0	50.0	63.9	63.9
	Bilongue II, Douala	26 (31)	100	80.6	19.4	19.4
	Bingomo, East	37 (53)	37.7	20.8	41.5	41.5
	Founaguedj, Far North	27 (42)	11.6	37.2	83.7	76.7
	Kombi III, Littoral	30 (44)	97.7	75.0	27.3	27.3
	Wouro Lawan, North	26 (40)	0.0	100	100	100
	Ntambeng II, Northwest	15 (28)	100	0.0	67.9	67.9
	Ngomeden, South	2 (6)	100	16.7	100	100
	Ngeme, Southwest	11 (30)	100	0.0	60.0	60.0
	Bachepang, West	33 (51)	100	78.4	27.5	23.5
	Djoungolo I, Yaoundé	11 (16)	100	75	37.5	37.5
	Total	256 (412)	62.0	50.6	56.4	55.2

^1^All values are percentages. No wheat flour samples were available from Ngomeden, South region.

### Public sector inputs, processes, and outputs

**FIGURE 2:**
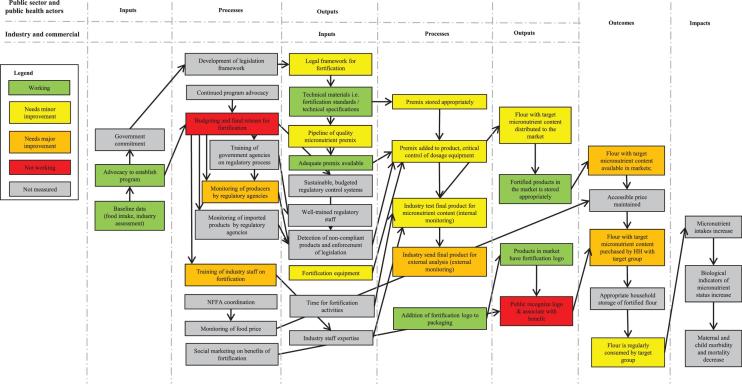
PIP results for Cameroon's large-scale edible oil fortification program in 2016. Box colors indicate program performance. HH, household; NFFA, National Food Fortification Alliance; PIP, program impact pathway.

**FIGURE 3:**
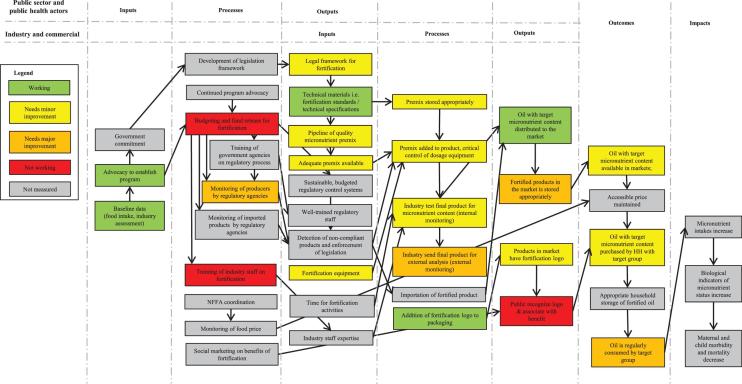
PIP results for Cameroon's large-scale wheat flour fortification program in 2016. Box colors indicate program performance. HH, household; NFFA, National Food Fortification Alliance; PIP, program impact pathway.

Baseline data, advocacy, and government commitment were identified as key initial inputs for establishment of both the oil and the flour fortification programs (**Figures 2** and **3**). Advocacy for large-scale fortification began in 2008, coinciding with a grant focused on staple food fortification from the Michael and Susan Dell Foundation to Helen Keller International. In 2008 the National Food Fortification Alliance (NFFA; in Cameroon, the Comité Technique National de Pilotage et de Coordination pour la Fortification Alimentaire en Micronutriments) was launched, and a national micronutrient survey was undertaken the following year. In 2011 legislation mandating the fortification of edible oil and wheat flour with micronutrients was instituted (Arrêté conjoint 2366/MINSANTE/MININDT/MINCOMMERCE du 24 août 2011 and Arrêté conjoint 2369/MINSANTE/MINIMIDT/MINCOMMERCE du 24 août 2011). Standards for wheat flour fortification and refined oil fortification have been published by the National Norms Agency and signed by 3 ministries: Public Health, Industry, and Trade. Thus, advocacy for establishing the program was successful in spurring development of a legal framework and technical materials for fortification, assistance in procuring equipment, and initial training of industry staff and national regulatory staff.

Although we did not formally collect data on current advocacy efforts, NFFA activity, and regulatory monitoring, stakeholder interviews indicated limited NFFA activity and advocacy in recent years and no regular disbursement of funds for fortification program support. The most recent program-monitoring plan spanned the period of 2013–2015 and stipulated 6-monthly regulatory monitoring at industry sites and markets; however, no routine regulatory monitoring was conducted by regulatory agencies in the 12 mo preceding the study.

### Wheat flour PIP

#### Wheat flour program inputs and processes from the industry perspective

A certificate of quality for the previous premix delivery was available for 70% (7/10) of flour millers (Table 2). The majority (8/10) of factories had an automated system for adding and mixing the micronutrient premix; the remaining factories used a manual premix addition process. All had the capacity to conduct qualitative tests for iron (i.e., a spot test), but equipment for conducting in-house quantitative assessments of the final product was available for only 1 of 10 factories (Table 2). Half (5/10) of the wheat flour producers cited challenges with premix procurement; of these, 2 cited cost as a major barrier, 2 noted logistical challenges (e.g., the importation of premix), and 1 mentioned inability to assess premix quality.

#### Wheat flour program outputs

Across all clusters, 58% of wheat flour samples collected from markets were found to be exposed to direct sunlight and 86% were exposed to air (Table 3). Overall, 94% of samples were both in their original packaging and labeled as fortified. In the household survey, 8.3% (SD = 6.4%) of respondents had heard of wheat flour fortification.

**FIGURE 4:**
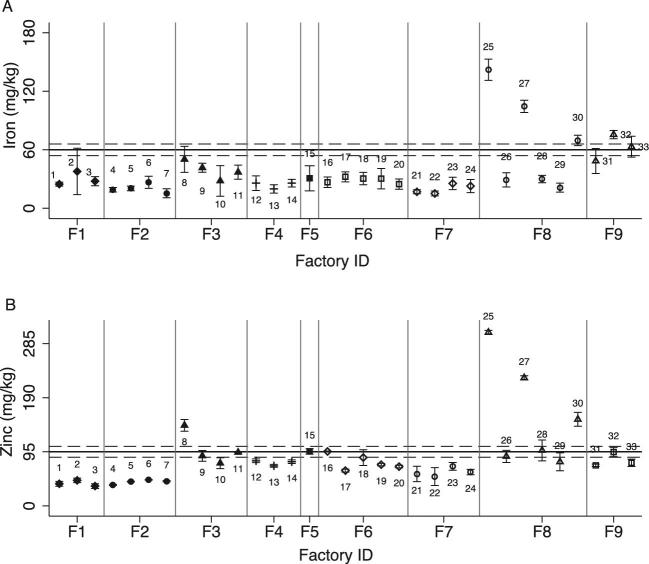
Total mean iron and zinc content of brand composite samples of wheat flour brands collected from industry sites, measured in triplicate. Error bars = 1 SD. (A) Total iron content of industry wheat flour samples. (B) Total zinc content of industry wheat flour samples. Numbers reflect the brands; gray vertical lines separate the different factories. The black line represents the fortification standard, and the gray dashed reference lines represent the lower and upper standard thresholds of the standard.

Iron and zinc content varied by brand but was generally below the standard for both nutrients (**Figure 4**). The mean total iron content of composite samples from factories was 37.6 mg/kg (95% CI: 28.0, 47.1 mg/kg), 63% of the national standard for iron, and the mean total zinc content of the samples was 85.2 mg/kg (95% CI: 65.9, 104 mg/kg), or 90% of the national standard for zinc (*P* < 0.0001 for difference in percentage of national standard achieved for iron compared with zinc). The percentage of the national standard achieved fell further when accounting for intrinsic iron and zinc content of wheat flour (**Table 4**). Assuming intrinsic iron and zinc content of 12.5 and 8.5 mg/kg, respectively, the mean estimated amounts of added minerals represent 42% of the target for iron and 81% of the target for zinc. No industry samples had iron or zinc concentrations below the assumed intrinsic iron and zinc concentrations (i.e., all industry samples were likely to have been fortified).

**TABLE 4:** Micronutrient levels of wheat flour and oil samples collected at industry, market, and household sites and the percentage of the target achieved

	Wheat flour^1^		Wheat flour^1^^,^^2^		Refined vegetable oil^1^
	Total iron	Total zinc	Added iron	Added zinc	Vitamin A
Factories					
*n*	33	33	33	33	12
Mean micronutrient content, mg/kg (95% CI)	37.6 (28.0, 47.1)	85.2 (65.9, 104)	25.1 (15.5, 34.6)	76.7 (57.4, 96.0)	16.9 (13.8, 20.0)
Mean micronutrient content as a percentage of national standard (95% CI)	63 (47, 78)	90 (70, 109)	42 (26, 58)	81 (60, 101)	141 (116, 167)
Median micronutrient content, mg/kg (IQR)	28.1 (13.0)	75.0 (37.0)	16.2 (13.0)	66.8 (37.9)	17.5 (7.8)
Median micronutrient content as a percentage of national standard (IQR)	47 (22)	79 (40)	27 (22)	70 (40)	146 (65)
No. of composite samples within the tolerable range (%)^3^	5/33 (15%)	9/33 (27%)	3/33 (9%)	9/33 (27%)	6/12 (50%)
Markets					
*n*	45	45	45	45	432
Mean micronutrient content, mg/kg (95% CI)	27.2 (22.1, 32.2)	46.8 (38.0, 55.5)	14.7 (9.6, 19.7)	38.3 (29.6, 47.0)	9.0 (7.4, 10.7)
Mean micronutrient content as a percentage of national standard (95% CI)	45 (37, 54)	49 (40, 58)	24 (16, 33)	40 (31, 50)	75 (62, 89)
Median micronutrient content, mg/kg (IQR)	22.3 (9.8)	42.5 (49.4)	9.8 (9.8)	34.0 (49.4)	8.8 (7.9)
Median micronutrient content as a percentage of national standard (IQR)	37 (16)	45 (52)	16 (16)	36 (52)	73 (66)
No. of samples^1^ within the tolerable range (%)^3^	2/45 (4%)	3/45 (7%)	2/45 (4%)	1/45 (2%)	134/432 (31%)
Households					
*n*	15	15	15	15	208
Mean micronutrient content, mg/kg (95% CI)	19.8 (13.9, 25.8)	32.5 (18.4, 46.7)	7.9 (2.3, 13.6)	24.5 (10.6, 38.3)	9.0 (7.3, 10.8)
Mean micronutrient content as a percentage of national standard (95% CI)	33 (23, 43)	34 (19, 49)	13 (5, 23)	26 (11, 40)	75 (60, 90)
Median micronutrient content, mg/kg (IQR)	18.7 (13.3)	40.50 (40.2)	6.2 (12.2)	32.0 (39.8)	9.3 (8.9)
Median micronutrient content as a percentage of national standard (IQR)	31 (22)	43 (42)	10 (20)	34 (42)	78 (74)
No. of samples^1^ within the tolerable range (%)^3^	1/15 (7%)	1/15 (7%)	1/15 (7%)	1/15 (7%)	78/208 (38%)

^1^Wheat flour samples consist of composite samples representing each brand (factory samples) or each brand found in each cluster (market and household samples). Oil samples consist of composite samples representing each brand (factory samples) or individual samples collected from markets and households.

^2^Values represent added mineral content assuming intrinsic iron and zinc contents of 12.5 and 8.5 mg/kg, respectively.

^3^Samples were considered within the tolerable range if they fell within the range of the national standard. For vitamin A in oil, an additional overage allowance of 30% was applied to the midpoint of the national standard (12 mg/kg) to construct the tolerable range.

#### Wheat flour program outcomes

**FIGURE 5:**
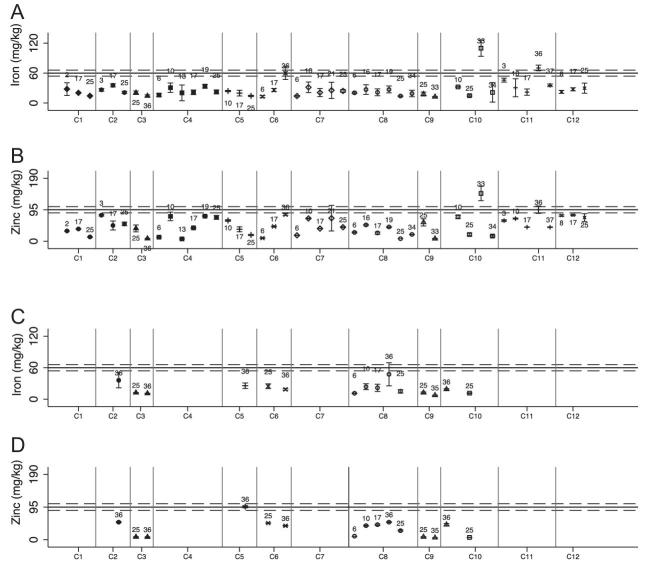
Total mean iron and zinc content of composite samples of wheat flour brands collected from markets and household, measured in triplicate. Error bars = 1 SD (accounting for analytical error). Vertical gray bars separate the clusters. Number labels reflect the brands. Horizontal gray reference lines represent the lower and upper acceptable thresholds, and the black line represents the national fortification standard. (A) Total iron concentration of wheat flour samples collected at market sites. (B) Total zinc concentration of wheat flour samples collected at market sites. (C) Iron concentration of wheat flour samples collected at household sites. (D) Zinc concentration of wheat flour samples collected at household sites.

Overall, the mean mineral content of composite samples did not differ among samples collected from markets compared with households for iron (market: 27.2 mg/kg, 95% CI: 22.1, 32.2 mg/kg; household: 19.8 mg/kg, 95% CI: 13.9, 25.8 mg/kg; *P* = 0.10) or zinc (market: 46.8 mg/kg, 95% CI: 38.0, 55.5 mg/kg; household: 32.5 mg/kg, 95% CI: 18.4, 46.7 mg/kg; *P* = 0.12), although there was variability by cluster and brand (Table 4, **Figure 5**). The mean iron content of pooled market and household samples was 25.3 mg/kg (95% CI: 21.2, 29.4 mg/kg), representing 42% of the target levels without adjusting for intrinsic iron, or 21% of the target levels after accounting for intrinsic iron. Mean iron content of pooled market and household samples was significantly lower than the mean iron content of industry samples (37.6 mg/kg, 95% CI: 28.0, 47.1 mg/kg; *P* = 0.007). The mean zinc content of pooled market and household samples was 43.2 mg/kg (95% CI: 35.8, 50.6 mg/kg), representing 45% of the target zinc content, or 37% of the target zinc content after accounting for intrinsic zinc. Mean zinc content of pooled market and household samples was also significantly lower than the mean zinc content of industry samples (85.2 mg/kg, 95% CI: 65.9, 104 mg/kg; *P* < 0.001).

The mean B vitamin content of composite samples from markets and households across all clusters was 1.79 mg/kg (SD = 0.64) for folic acid, 36% of the national standard, and 0.012 mg/kg (SD = 0.01) for vitamin B-12, 29% of the national standard (**Table 5**).

**TABLE 5:** B vitamin content of combined market and household samples within each cluster

Cluster, region	Folic acid (mg/kg)	Folic acid as percentage of national standard	Vitamin B-12 (mg/kg)	Vitamin B-12 as percentage of national standard
Sabongari, Adamaoua	0.785	16	0.0049	12
Nkolkougda I, Central	0.774	15	0.0029	7
Bilongue II, Douala	2.76	55	0.014	35
Bingomo, East	2.18	44	0.0056	14
Founaguedje, Far North	2.45	49	0.0127	32
Kombi III, Littoral	1.49	30	0.027	68
Wouro Lawan, North	1.83	37	0.0363	91
Ntambeng II, Northwest	1.85	37	0.0068	17
Ngomeden, South	1.98	40	0.0106	27
Ngeme, Southwest	1.88	38	0.0104	26
Bachepang, West	2.38	48	0.0069	17
Djoungolo I, Yaounde	1.11	22	0.0015	4
Overall mean (SD)	1.79 (0.64)	36 (13)	0.012 (0.01)	29 (26)

Reach and frequency of consumption of wheat flour consumption varied significantly by cluster (*P* < 0.001) (**Table 6**). Overall, reach was higher among children (mean: 86%; 95% CI: 80%, 91%) than among women (mean: 82%; 95% CI: 76%, 89%). Among individuals who had consumed wheat flour in the previous week, the mean frequency of weekly consumption for women was 7.1 (95% CI: 5.5, 8.6) and for children 9.0 (95% CI: 7.4, 10.6). Overall, 50% of all women and 64% of all children consumed fortifiable wheat flour 5 or more times per week.

**TABLE 6:** Proportion of Cameroonian women and children consuming refined vegetable oil and wheat flour and weekly frequency of intake among consumers by sentinel site^1^

	Sabongari, Adamaoua	Nkolkougda I, Central	Bilongue II, Douala	Bingomo, East	Founaguedje, Far North	Kombi III, Littoral	Wouro Lawan, North	Ntambeng II, Northwest	Ngomeden, South	Ngeme, Southwest	Bachepang, West	Djoungolo I, Yaoundé	Total
Women													
Wheat flour reach, %	87 (74, 95)	76 (63, 87)	88 (77, 95)	67 (53, 80)	68 (53, 80)	86 (73, 94)	88 (76, 95)	96 (87, 100)	87 (75, 95)	96 (86, 100)	69 (54, 81)	78 (65, 89)	82 (76, 89)
Consumers, *n*	41	39	51	34	34	43	44	50	47	48	33	38	502
Frequency wheat flour, times/week	6.5 (5.2, 7.8)	5.6 (3, 8.3)	8.8 (7, 10.6)	6.2 (4.1,8.3)	5.3 (4.3, 6.4)	6 (4.7, 7.4)	7.3 (6.4, 8.2)	8.5 (6.9, 10.2)	3.4 (2.8, 4)	8.9 (6.5, 11.3)	4.4 (3.6, 5.3)	12.8 (6.9, 18.7)	7.1 (5.5, 8.6)
Children													
Wheat flour reach, %	95 (84, 99)	80 (66, 90)	84 (72, 92)	76 (63, 87)	74 (60, 85)	90 (78, 97)	86 (73, 94)	100 (93, 100)	91 (80, 97)	100 (92, 100)	75 (60, 87)	82 (68, 91)	85 (80, 91)
Consumers, *n*	44	40	47	40	37	45	43	50	49	48	35	38	516
Frequency wheat flour, times/week	8.8 (7.2, 10.5)	7.4 (4.5, 10.3)	11.2 (9, 13.3)	8.7 (6.3, 11)	6.2 (4.8, 7.6)	8.2 (6.9, 9.4)	10.9 (9.2, 12.7)	11.5 (9.4, 13.7)	5.7 (4.7, 6.7)	11.5 (8.6, 14.4)	4.4 (3.8, 5.1)	12.3 (8.3, 16.3)	9.0 (7.4, 10.6)
Women													
Refined oil reach, %	98 (89, 100)	49 (35, 63)	66 (52, 78)	60 (45, 73)	76 (62, 87)	36 (23, 51)	60 (45, 74)	79 (65, 89)	46 (33, 60)	86 (73, 94)	23 (12, 37)	90 (79, 97)	64 (50, 78)
Consumers, *n*	46	23	38	31	38	17	30	41	24	43	11	44	386
Frequency refined oil, times/week	16.5 (13.1, 19.8)	8.4 (5.3, 11.4)	7.6 (6, 9.1)	9 (7.1, 10.9)	15.2 (13.3, 17.2)	6.6 (4.7, 8.5)	16.9 (13.7, 20.1)	10.1 (7.7, 12.5)	7.8 (5.1, 10.6)	9.5 (7.1, 11.9)	9.6 (2.6, 16.7)	6.5 (4.5, 8.5)	10.7 (8.0, 13.3)
Children													
Refined oil reach, %	70 (54, 83)	41 (27, 56)	55 (42, 69)	41 (28, 56)	42 (28, 57)	45 (31, 60)	48 (34, 63)	77 (63, 88)	39 (26, 53)	64 (49, 77)	21 (10, 35)	84 (70, , 93)	52 (40, 63)
Consumers, n	30	18	29	21	21	13	24	38	20	27	9	38	288
Frequency refined oil, times/week	15.4 (12.6, 18.2)	7.9 (3.9, 12)	7.2 (5.6, 8.9)	13.5 (9, 18)	15.5 (13.2, 17.8)	6.5 (3.8, 9.2)	16.5 (13.5, 19.5)	10.8 (8.3, 13.4)	8.1 (5.2, 10.9)	8.7 (6.3, 11.1)	8.8 (2.4, 15.2)	7.5 (4.7, 10.3)	10.7 (8.3, 13.1)

^1^Values are means (95% CIs); *n* = 613 women aged 15–49 y and 594 children aged 6 mo to 15 y. Weekly frequency of consumption is calculated only for those who reported consuming the selected food during the previous week.

### Refined vegetable oil

#### Oil program inputs and processes

Overall, 67% (6/9) of oil industries had the certificate of quality for the previous premix delivery. Documentation of flow rate monitoring was available from 67% (6/9) of oil refiners, and 56% (5/9) of oil factories had automated dosing and mixing equipment. Equipment for conducting in-house quantitative assessments of the final product was available at 33% (3/9) of oil refiners, whereas all factories had the capacity to conduct qualitative assessments for vitamin A (Table 2). Overall, 44% (4/9) of oil producers stated they had challenges related to premix procurement; of these, 75% (3/4) of challenges were related to premix costs, whereas the other 25% (1/4) were related to procurement logistics.

#### Oil program outputs

Of the oil samples collected at markets, 62% were exposed to direct sunlight, 51% were exposed to air, 56% were in their original packaging, and 55% had the fortification logo present on the packaging (Table 3). Just 14% of respondents had heard of refined oil fortification.

**FIGURE 6:**
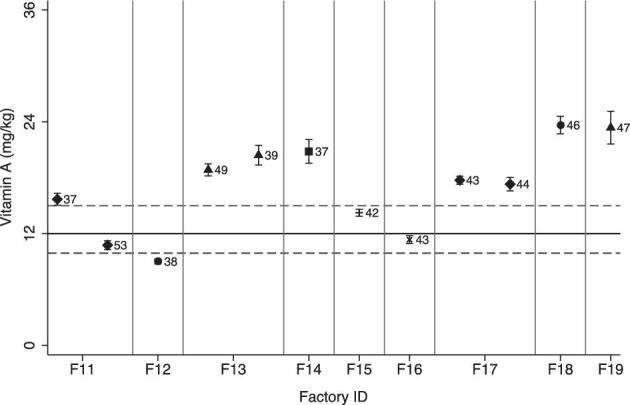
Mean vitamin A content of composite samples of refined vegetable oil brands. Error bars = 1 SD. Numbers reflect the brands; gray vertical lines separate the different factories. The solid black line represents the fortification standard, and the gray dotted reference lines represent the lower and upper standard thresholds of the standard.

The mean vitamin A content of industry samples was 16.9 mg/kg (95% CI: 16.8, 20.0 mg/kg), representing 141% of the national standard. Overall, 50% (6/12) of the individual industry samples were within the tolerable range of the standard, and 5 of the 12 samples (42%) had a vitamin A content >30% above the national standard (Table 4, **Figure 6**).

#### Oil program outcomes

**FIGURE 7:**
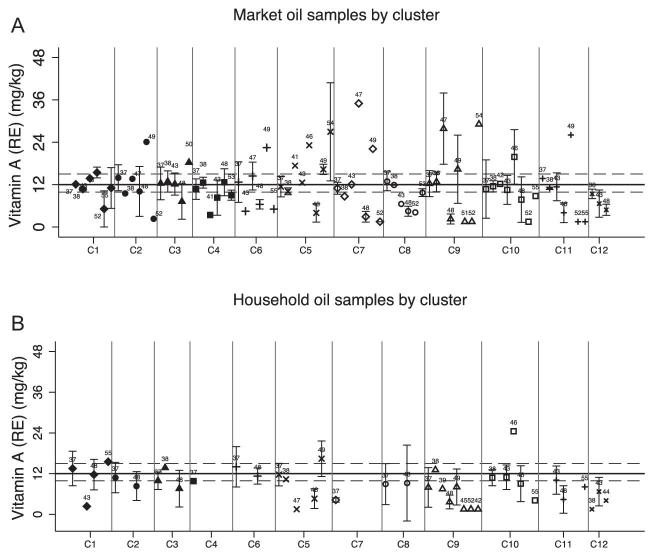
Mean vitamin A content of composite samples of refined oil brands collected at markets and households within each cluster. Error bars = 1 SD (accounting for variability in individual samples and analytical error). Vertical gray bars separate the clusters. Number labels reflect the brands. Horizontal gray reference lines represent the lower and upper acceptable thresholds, and the black line represents the national fortification standard. (A) Vitamin A concentration of refined oil samples collected at market sites. (B) Vitamin A concentration of refined oil samples collected at household sites.

The vitamin A content of samples collected from markets (9.0 mg/kg; 95% CI: 7.4, 10.7 mg/kg) and households (9.0 mg/kg; 95% CI: 7.3, 10.8 mg/kg) was not significantly different (*P* = 0.99) (Table 5). The mean vitamin A content of pooled market and household samples was 9.0 mg/kg (95% CI: 7.8, 10.3 mg/kg), which was significantly lower than the mean vitamin A content of industry samples (16.9 mg/kg; 95% CI: 16.8, 20.0 mg/kg, *P* = < 0.001). Vitamin A concentration of composite oil samples varied among sentinel sites and by brand (**Figure 7**).

For both women and children, coverage of refined oil consumption differed significantly among clusters (*P* < 0.001 for both groups) (Table 6). Overall, reach was higher among women (mean: 64%; 95% CI: 50%, 78%) than among children (mean: 52%; 95% CI: 40%, 63%). The mean frequency of consumption of refined vegetable oil among individuals who consumed the food in the previous week was 10.7 (95% CI: 8.0, 13.3) for women and 10.7 (95% CI: 8.3, 13.1) for children. Overall, 37% of all women and 38% of all children consumed refined vegetable oil ≥5 times per week.

## Discussion

We used a PIP approach to conduct a detailed evaluation of the operations of the mandatory national oil and wheat flour fortification programs in Cameroon. Strong external financing and technical support characterized the launch of the program; however, in recent years there has been a lack of strong leadership to organize coordination activities and to budget for the program, which has stalled government communication and monitoring activities. Nevertheless, the performance of the oil fortification program appears to have improved, with the majority of oil samples containing vitamin A at levels ∼75% of the national standard compared to ∼44% in Yaoundé and Douala in 2012 (25). The lower vitamin A level of household and market samples relative to industry samples possibly indicates vitamin A loss due to storage and handling conditions, dilution with unfortified oil from other sources in the market, and/or biased sampling at the industry level, where composite samples were prepared based on a convenience sample of brands and batches available at the time of the scheduled visit. Support for premix procurement and management, external quality control, and further monitoring of imported oils were noted as opportunities to further strengthen the oil fortification program.

Although all wheat flour millers appeared to be distributing fortified products, the wheat flour fortification levels were below the target even at the factory level (with the exception of a few samples with high mineral concentrations, which may reflect inadequate mixing of the product). This situation was likely undetected due to the reliance by millers on qualitative testing for internal monitoring and lack of access to external laboratories for quantitative measures of micronutrient content. Factory records appear to indicate adequate premix availability on average, yet gaps in premix management practices and fortification-specific training may contribute to this discrepancy.

The impact of a food fortification program is a function of *1*) program reach among population groups at risk of deficiency, *2*) consumption by the target population of sufficient quantities on a regular basis, and *3*) adequate fortification of target products. We observed that the average reach of wheat flour products was >80% across the sentinel sites for both women and children, consistent with that observed in the previous national survey (21). Although both the reach of wheat flour and the frequency of intake among consumers would be adequate to expect some improvement in micronutrient intake, the low levels of wheat flour fortification observed suggest limited impact on micronutrient status at the population level, particularly in areas with less frequent consumption of wheat flour products.

In a previous evaluation of the program, no change in vitamin A status was observed post-fortification in Yaoundé and Douala, possibly because only ∼44% of oil samples were fortified (25). The results of the current study suggest greater potential for impact because the vitamin A content of oil samples was closer to the target levels (on average, ∼75% of target vitamin A levels). On the other hand, the variability in reach of refined, “fortifiable” oil (21), as well as the regional variation in prevalence of vitamin A deficiency (21, 29), suggests variability in the potential for impact; improvements in vitamin A status are most likely in areas where vitamin A deficiency and fortified oil consumption are both common. Confirmation that the current oil fortification levels have been sustained or increased would help justify an impact evaluation to assess change in biomarkers of vitamin A status.

A principle limitation of this study is that by taking a cross-sectional approach, we may have missed important fluctuations over time in program operation, particularly in the concentrations of micronutrients in foods. Ideally, a continuous monitoring system would be in place to provide routine information on the micronutrient content of foods in the selected sentinel sites, as used in Costa Rica (30), but this was not feasible in the context of this project. However, by using a longer (>1 y) recall period for some industry-level variables, including availability of premix quality certificates and premix costs, we were able to capture an average value for practices that may vary over time. The random walk methodology used in the household survey also has limitations with regard to obtaining a representative sample of households, but it was used to balance the availability of resources and minimize burden on the data collection team. If new monitoring initiatives include routine sentinel site monitoring, we recommend conducting a census within each cluster and using this list to randomly select households.

We were successful in gathering data from the majority of oil and wheat flour factories, including the producers with the highest market share. However, it was not possible to gather quantitative information on the market share of each brand; thus, it was not possible to weight the quantitative results accordingly. This limitation would be expected to underestimate the performance of the program because larger industries with greater market share may have more resources available to ensure their products are meeting the national standard. In selecting the clusters for the market and household data collection, emphasis was placed on having wide geographical representation, but we emphasize that the results reflect the situation in the 12 sentinel sites and are not meant to be regionally or nationally representative. This approach, however, is generally more feasible from both financial and operational perspectives compared with large-scale representative surveys. This sentinel site monitoring could be repeated to track changes over time to provide information about the program for decision-making.

These results have several implications for the fortification program in Cameroon. Although early advocacy efforts were successful in establishing the program, awareness among several stakeholders’ groups, namely parliamentarians and legislators, remains limited. This has implications for numerous aspects of the program, especially funding for activities to be conducted by public institutions, such as a sustained regulatory system. Strengthening advocacy efforts to ensure all stakeholders are aware of the program and understand their specific role within it would be a prerequisite to delivering long-term commitment and adequate funding. Coordination efforts can also be strengthened and accountability mechanisms established to ensure stakeholders are fulfilling their roles within the program. The establishment of a single repository of monitoring reports conducted by various control bodies may also be a valuable resource to ensure effective coordination and knowledge sharing among stakeholders.

Interviews with both industries, but particularly wheat flour producers, revealed considerable financial pressure related to fortification. This observation is consistent with a survey of respondents from 13 countries in which the price of premix was identified as the major barrier to adequate fortification (31). In Cameroon, both edible oil and wheat flour are price-controlled commodities, so industries have limited leeway to pass along the cost of premix and related taxes to the consumer. At the same time, it is important for the success of the program that adequately fortified products remain affordable to consumers. Ensuring that the cost increases arising from fortification are acceptable to both industries and consumers is central to ensuring the long-term sustainability of the program. We suggest that steps such as the introduction of a waiver on premix taxes could potentially relieve some of this pressure and encourage factories to invest elsewhere in ensuring that products are adequately fortified, while potentially helping protect local factories from external competition. These observations also highlight the importance of continuous communication among all stakeholders of the program through routine NFFA meetings to review information such as food prices and external monitoring results.

Our analysis indicated that there is little recent external quality control monitoring of the program, in terms of both regulatory monitoring by government agencies and the use of external laboratories by industries. Identifying external laboratories that can provide timely results was a challenge noted by many producers. Whereas there was capacity for quantitative analysis (the iCheck instrument) at some oil factories, only 1 wheat flour miller reported internal access to quantitative measures, and reported external analyses of wheat flour products were rare. With only iron spot tests to assess the product quality, the low fortification levels in wheat flour were undetected until this study. Efforts to support the program should include restoring the capacity of an external laboratory to conduct routine quantitative testing of fortified food samples, as well as establishing a system for collecting samples and relaying results, with clear roles and accountability for all partners.

Although public recognition and knowledge of fortified products were very low for both food vehicles, the additional impact of social marketing is unclear in the Cameroon context of high baseline reach of oil and wheat flour and mandatory fortification. Similarly, low recognition of wheat flour fortification was observed in Yaoundé and Douala in 2012, yet substantial increases in micronutrient status were observed (26). Thus, despite the salience of this element within the PIP, in Cameroon program funds are likely better invested in addressing other identified barriers, such as regulatory monitoring and increasing access to external reference laboratories. Social marketing investments are likely to be more important in a setting with voluntary fortification, in which consumers have a choice between fortified and unfortified products (potentially with cost implications).

Program impact pathways have been used in diverse contexts to conceptualize the intended causal pathways that a nutrition intervention is designed to affect and to provide a framework for identifying data needs for program monitoring and evaluation (13–17). An advantage of the PIP approach is that it forces the user to explicitly define the assumptions about how the program is expected to work. Once a PIP is developed for a specific program, the results can be continuously updated as new monitoring data become available, and the PIP can be applied periodically to indicate areas of special focus for routine monitoring. The PIP can thus be integrated with other approaches for collection of monitoring data, including routine administrative reviews and population surveillance (19, 20). The anticipated uses for the PIP developed for Cameroon are to *1*) illustrate to stakeholders where their activities fit within the broader program and *2*) define indicators to be monitored by NFFA.

Through this analysis, we identified several methodological considerations for the use of a PIP for fortification program monitoring. First, it is important to have clear definitions for each of the indicators to be tracked and particularly to specify whether these definitions are applied at the industry level or the individual factory level; for example, *1*) premix purchased by a specific factory as a percentage of that needed for the annual production by that factory, *2*) total premix purchased by all domestic factories as a percentage of that needed for total domestic production, or *3*) proportion of factories with sufficient premix purchased in relation to their production. In addition, it is useful to specify the criteria for adequate performance in advance, where possible, to avoid influence of knowledge of the results on these thresholds.

In conclusion, the PIP approach requires the user to explicitly define the expected causal pathways whereby a nutrition intervention program will have the intended effects. Application of this process for fortification programs in Cameroon identified opportunities to strengthen the program to fortify refined vegetable oil and wheat flour. Vitamin A concentrations in oil increased in comparison to previous monitoring data, although they were still below national norms. On average, micronutrients in wheat flour were substantially less than targets, indicating the need for further monitoring and corrective action. Support for premix procurement and management, external quality control and quantitative micronutrient analysis, and stronger monitoring of imported oils offer opportunities to further strengthen the potential impact of the program on micronutrient status and health. It is expected that this PIP approach will help stakeholders identify more systematically all the key barriers to program performance and to guide decision-making and action. Using an abridged version of the PIP to reassess factors identified as needing major improvement would be a logical next step, while gaining further insights from stakeholders on the value of the PIP approach for guiding decision-making.

## Supplementary Material

nzz076_Supplemental_Files

## Notes

This study was supported by a grant from Sight and Life to the University of California, Davis. The funder had no role in the study design, implementation, analysis, or decision to publish.

Author disclosures: HEM worked as a consultant for Sight and Life, which provided the funds for this study, in the 3 y preceding the research. JGA, HL, MN, AN, IT, AT, AP, DWK, KHB, and RE-S, no conflicts of interest.

Supplemental Tables 1–3 are available from the “Supplementary data” link in the online posting of the article and from the same link in the online table of contents at https://academic.oup.com/cdn/.
