# Supplementary material for: Monitoring of the National Oil and Wheat Flour Fortification Program in Cameroon Using a Program Impact Pathway Approach
Source: Curr Dev Nutr. 2019 Jun 20;3(8):nzz076. doi: 10.1093/cdn/nzz076 (PMC6660062; doi:10.1093/cdn/nzz076)
Supplement: nzz076_Supplemental_Files [file nzz076_supplemental_files.zip › S1.pdf]

Online Supporting Material

Actors	Group	Box	Description	Indicators	Data source	Criteria for 'success' (working, minor needs improvement, needs significant improvement, not working, not measured)		Result	Outcome
Public sector and public health actors	Inputs	1	Government commitment	Not measured					
Public sector and public health actors	Inputs	2	Advocacy to establish programme	Awareness of the fortification programme amongst relevant organisations and institutions	Communication with key stakeholders	Working	All relevant stakeholders are aware of the fortification programme and its objectives	All relevant stakeholders are aware of program and its objectives	Working
						Needs minor improvement	Some but not all relevant stakeholders are aware of the fortification programme and its objectives		
						Needs major improvement	Only a small number of relevant stakeholders are aware of the fortification programme and its objectives		
						Not working	None of the major stakeholders are aware of the fortification programme and its objectives		
Public sector and public health actors	Inputs	3	Baseline data (food intake, industry assessment)	Availability of biomarker data for assessing deficiency prevalence and dietary data for modelling fortification levels (at the time of program advocacy/implementation)	Not measured	Working	Current information available on dietary intake, micronutrient status, industry assessment	Information on individual dietary intake and micronutrient status biomarkers collected prior to program start; industry assessment conducted	Working
						Needs minor improvement	Information available, but 5+ years old		
						Needs major improvement	Only partial information available		
						Not working	No data available		
Public sector and public health actors	Process	1	Development of legal framework for fortification	Not measured					

Online Supporting Material

Public sector and public health actors	Process	2	Continued program advocacy	Not measured					
Public sector and public health actors	Process	3	Budgeting and fund release for fortification	Funding for routine monitoring of the program	Communication with key stakeholders	Working	Adequate funding for external monitoring in government budget and funds released to relevant agency / ministry.	No monitoring funds budgeted	Not working
						Needs minor improvement	Above, but funds are less than desired or released inconsistently		
						Needs major improvement	Funds allocated but not released		
						Not working	No monitoring funds budgeted		
Public sector and public health actors	Process	4	Training of government agencies on regulatory process	Not measured					
Public sector and public health actors	Processes	5	Monitoring of producers by regulatory agencies	Last time regulatory agencies conducted monitoring	Communication with key stakeholders	Working	Regular monitoring of fortification programme at industry level (at least quarterly)	Monitoring plans for factory level exist but no monitoring conducted in the past 12 months	Needs major improvement
						Needs minor improvement	Irregular monitoring of fortification programme at industry level (at least annually)		
						Needs major improvement	Monitoring plans for factory level but no monitoring conducted in the past 12 months		
						Not working	No monitoring plans for factory level within the regulatory agencies		

Public sector and public health actors	Process	6	Monitoring of imported products by regulatory agencies	Not measured					
Public sector and public health actors	Process	7	Training of industry staff on fortification	Support for training / capacity building	Industry questionnaire and monitoring visit observations	Working	Staff at all major industries received training in the past 18 months	Staff at 20% of industries provided training on quality control in past 18 months	Needs major improvement
						Needs minor improvement	Staff at over 60% of industries have received training in the last 18 months		
						Needs major improvement	Staff at 20-60% of industries have received training in the last 18 months		
						Not working	In less than 20% of industries staff have not received training in the past 18 months		
Public sector and public health actors	Process	8	NFFA coordination	Not measured					
Public sector and public health actors	Process	9	Monitoring of food prices	Not measured					
Public sector and public health actors	Process	10	Social marketing on benefits of fortified flour	Not measured					
Public sector and public health actors<br><br>Industry and Commercial sector	Output<br><br>Input	1	Legal framework for fortification	Existence of fortification legislation and standards	Copies of government documents	Working	Framework in place that provides adequate legislative powers for action to be taken against non-compliant producers	Framework in place but needs updating	Needs minor improvement
						Needs minor improvement	Framework in place but needs updating to ensure adequate powers to act against non-compliant producers		
						Needs major improvement	Framework in place but not adequate to provide legislative powers to act against non-compliers		
						Not working	No framework in place		

Public sector and public health actors<br><br>Industry and Commercial sector	Output<br><br>Input	2	Technical specifications on fortification	Existence of technical specifications published by relevant government body	Copies of government documents	Working	Fortification standards for wheat flour exist and are up to date	Standards exist and are in the public domain	Working
						Needs minor improvement	Fortification standards for wheat flour exist and are up to date but need updating		
						Needs major improvement	Fortification standards for wheat flour are in development phase or finalised but not available to relevant stakeholders		
						Not working	Documents do not exist and are not being developed		
Public sector and public health actors<br><br>Industry and Commercial sector	Output<br><br>Input	3	Pipeline of quality micronutrient premix	Availability of premix quality certificates	Industry questionnaire and monitoring visit observations	Working	More than 80% of factories have premix certificate of quality or premix analysis	70% of factories have permix certificate of quality	Needs minor improvement
						Needs minor improvement	50-80% of factories have certificate of premix analysis		
						Needs major improvement	20-49% of factories have certificate of premix analysis		
						Not working	Less than 20% of factories have certificate of premix analysis		
Public sector and public health actors<br><br>Industry and Commercial sector	Output<br><br>Input	4	Adequate quantity of premix available	Median premix available across all factories as percentage required for reported production	Industry questionnaire and monitoring visit observations	Working	The volume of premix equates to more than 80% of final product volume and no stock outs in past 12 months	Premix procured in 2015 adequate to meet 111% of 2015 product volumes & no stock outs	Working
						Needs minor improvement	Volume of premix equates to between 50-80% of final product volume and no stock outs in past 12 months		
						Needs major improvement	Volume of premix equates to between 20-49% of final product volume		
						Not working	Volume of premix equates to <20%		
Public sector and public health actors<br><br>Industry and Commercial sector	Output<br><br>Input	5	Sustainable, budgeted regulatory control system	Not measured					

Public sector and public health actors<br><br>Industry and Commercial sector	Output<br><br>Input	6	Well trained regulatory staff	Not measured					
Public sector and public health actors<br><br>Industry and Commercial sector	Output<br><br>Input	7	Detection of non-compliant products and enforcement of legislation	Not measured					
Public sector and public health actors<br><br>Industry and Commercial sector	Output<br><br>Input	8	Fortification equipment	Percentage of factories with automated dosing and mixing system for fortificants	Industry questionnaire and monitoring visit observations	Working	> 80% of factories have automated premix dosing and mixing equipment	80% of factories with automated dosing and mixing system for fortificants	Needs minor improvement
						Needs minor improvement	50-80% of factories have automated premix dosing and mixing equipment		
						Needs major improvement	20-49% of factories have automated premix dosing and mixing equipment		
						Not working	Less than 20% of factories have automated premix dosing and mixing equipment		
Public sector and public health actors<br><br>Industry and Commercial sector	Output<br><br>Input	9	Time for fortification activities	Not measured					

Online Supporting Material

Public sector and public health actors<br><br>Industry and Commercial sector	Output<br><br>Input	10	Industry staff expertise	Not measured					
Industry and Commercial sector	Process	1	Premix stored appropriately	Premix is stored in appropriate conditions	Industry questionnaire and monitoring visit observations	Working	More than 80% of factories score 4-5	60% of factories have appropriate premix storage	Needs minor improvement
						Needs minor improvement	50-80% of factories score 4-5		
						Needs major improvement	20-49% of factories score 4-5		
						Not working	Less than 20% of factories score 4-5		
Industry and Commercial sector	Process	2	Premix added to product, critical control of dosage equipment	Records of flow rate are recorded	Industry questionnaire and monitoring visit observations	Working	More than 80% of factories have evidence that flow rate checked regularly	70% of factories had evidence of flow rate checking available	Needs minor improvement
						Needs minor improvement	50-79% of factories have evidence that flow rate is checked regularly		
						Needs major improvement	20-49% of factories have evidence that evidence that flow rate is checked regularly		
						Not working	Less than 20% of factories have evidence that evidence that flow rate is checked regularly		
Industry and Commercial sector	Process	3	Industry test final product for micronutrient content (internal monitoring)	In house quality control machines are available and utilised regularly on final product	Industry questionnaire and monitoring visit observations	Working	>80% of industries have equipment to conduct quantitative analysis, reagents are available	100% of factories have equipment and reagents to conduct *qualitative* analysis	Needs minor improvement
						Needs minor improvement	>80% of factories have some form of test method (qualitative and quantitative)		
						Needs major improvement	Equipment and reagents currently not available but evidence of some previous testing		
						Not working	Less than 20% of factories have equipment to conduct analysis		

Online Supporting Material

Industry and Commercial sector	Process	4	Industry send final product for external analysis (external monitoring)	Factory sends samples for external lab assessment	Industry questionnaire and monitoring visit observations	Working	>80% of industries have conducted external laboratory assessment in the past 18 months	30% of factories have conducted external laboratory assessment in the past 18 months	Needs major improvement
						Needs minor improvement	50-80% of industries have conducted external laboratory assessment in the past 18 months		
						Needs major improvement	20-49% of industries have conducted external laboratory assessment in the past 18 months		
						Not working	<20% of industries have conducted external laboratory assessment in the past 18 months		
Industry and Commercial sector	Process	5	Addition of fortification logo to packaging	Fortification logo on final product	Industry questionnaire and monitoring visit observations	Working	>80% labels have logo	100% of products at factory have the fortification logo	Working
						Needs minor improvement	50-80% labels have logo		
						Needs major improvement	20-49% labels have logo		
						Not working	<20% labels have logo		
Industry and Commercial sector	Outputs	1	Flour with target micronutrient content distributed to the market	Mean iron content of flour leaving factories as percentage of national standard	Analysis of samples collected at industry, market or household	Working	Mean iron concentration of flour samples from industry is >80% of national standard	Mean iron concentration of flour samples from industry is 63% of national standard	Needs minor improvement
						Needs minor improvement	Mean iron concentration of flour samples from industry is 50-80% of national standard		
						Needs major improvement	Mean iron concentration of flour samples from industry is 20-49% of national standard		
						Not working	Mean iron concentration of flour samples from industry is <20% of national standard		
Industry and Commercial sector	Outputs	2	Flour in market is stored correctly	Flour in market is in original packaging	Market sample collection	Working	>80% in original packaging	94% of products at market level in original packaging	Working
						Needs minor improvement	50-80% in original packaging		
						Needs major improvement	20-49% in original packaging		
						Not working	<20% in original packaging		
Industry and Commercial sector	Outputs	3	Product in market have fortification logo	Product has fortification logo	Market sample collection	Working	>80% labels have logo	94% of products at market level have fortification logo	Working
						Needs minor improvement	50-80% labels have logo		
						Needs major improvement	20-49% labels have logo		
						Not working	<20% imported flour fortified in target range		

Online Supporting Material

Industry and Commercial sector	Outputs	4	Public recognizes logo, understands link between fortified product and health	Target groups have heard of fortified flour	Household questionnaire	Working	>80% have heard about fortified flour and know it is good for health	8% of population have heard about fortified flour	Not working
						Needs minor improvement	50-80% have heard about fortified flour and know it is good for health		
						Needs major improvement	20-49% have heard about fortified flour		
						Not working	<20% have heard about fortified flour		
Public sector and public health actors<br><br>Industry and Commercial sector	Outcomes	1	Flour with target micronutrient content available in market	Mean iron content of flour samples collected at markets as percentage of national standard	Analysis of samples collected at industry, market or household	Working	Mean iron concentration of flour samples from markets is >80% of national standard	Mean iron concentration of flour samples from markets was 45% of national standard	Needs major improvement
						Needs minor improvement	Mean iron concentration of flour samples from markets is 50-80% of national standard		
						Needs major improvement	Mean iron concentration of flour samples from markets is 20-49% of national standard		
						Not working	Mean iron concentration of flour samples from markets is <20% of national standard		
Public sector and public health actors<br><br>Industry and Commercial sector	Outcomes	2	Maintained accessible price for fortified flour	Not measured					
Public sector and public health actors<br><br>Industry and Commercial sector	Outcomes	3	Flour with target micronutrient content purchased by HH with target group	Mean iron content of flour samples collected at households as percentage of national standard	Analysis of samples collected at industry, market or household	Working	Mean iron concentration of flour samples from households is >80% of national standard	Mean iron concentration of flour samples from households was 33% of national standard	Needs major improvement
						Needs minor improvement	Mean iron concentration of flour samples from households is 50-80% of national standard		
						Needs major improvement	Mean iron concentration of flour samples from households is 20-49% of national standard		
						Not working	Mean iron concentration of flour samples from households is <20% of national standard		

Public sector and public health actors<br><br>Industry and Commercial sector	Outcomes	4	Appropriate household storage of flour	Not measured					
Public sector and public health actors<br><br>Industry and Commercial sector	Outcomes	5	Flour is regularly consumed by target group	Flour consumption by target groups	Household questionnaire	Working	>80% of the population consume fortifiable flour more than 5 time a week	50% of women of reproductive age consumed wheat flour more than 5 times per week	Needs minor improvement
						Needs minor improvement	50-80% of the population consume fortifiable flour more than 5 time a week		
						Needs major improvement	20-49% of the population consume fortifiable flour more than 5 time a week		
						Not working	<20% of the population consume fortifiable flour more than 5 time a week		
Public sector and public health actors<br><br>Industry and Commercial sector	Impact	1	Micronutrient intake increases among target group	Not measured					
Public sector and public health actorsIndustry and Commercial sector	Impact	2	Biological indicators of zinc, iron, folate, and B12 status increase	Not measured					
Public sector and public health actors<br><br>Industry and Commercial sector	Impact	3	Maternal and child morbidity and mortality decrease	Not measured					
