# Supplementary material for: Monitoring of the National Oil and Wheat Flour Fortification Program in Cameroon Using a Program Impact Pathway Approach
Source: Curr Dev Nutr. 2019 Jun 20;3(8):nzz076. doi: 10.1093/cdn/nzz076 (PMC6660062; doi:10.1093/cdn/nzz076)
Supplement: nzz076_Supplemental_Files [file nzz076_supplemental_files.zip › S3_Number_of_samples_in_each_brand-cluster_composite.pdf]

Online Supporting Material

Data collection site	Cluster	Brand	Number of samples per composite
household	33	M:30	1
household	33	M:99	1
household	41	M:99	1
household	48	M:30	1
household	48	M:99	1
household	54	M:41	1
household	54	M:99	1
household	55	M:29	1
household	55	M:30	1
household	63	M:30	1
household	20	M:99	2
household	54	M:01	2
household	54	M:30	2
household	54	M:34	3
household	55	M:99	6
market	1	M:40	1
market	37	M: 01	1
market	37	M:34	1
market	37	M:38	1
market	52	M:46	1
market	54	M:07	1
market	54	M:30	1
market	63	M:24	1
market	63	M:28	1
market	63	M:41	1
market	77	M:88	1
market	77	M:99	1
market	89	M:34	1
market	20	M:39	2
market	33	M:99	2
market	37	M:10	2
market	48	M:99	2
market	54	M:01	2
market	54	M:10	2
market	55	M:24	2
market	52	M:30	3
market	54	M:28	3
market	54	M:34	3
market	77	M:41	4
market	37	M:41	5
market	52	M:01	5

market	77	M:39	5
market	41	M:34	7
market	41	M:41	7
market	52	M:34	7
market	52	M:41	7
market	89	M:44	7
market	33	M:30	8
market	1	M:30	9
market	20	M:34	9
market	41	M:30	9
market	1	M:34	10
market	48	M:01	10
market	55	M:30	12
market	20	M:30	13
market	48	M:34	13
market	37	M:30	16
market	63	M:30	24
market	89	M:30	24
market	77	M:34	28
