# Supplementary material for: Monitoring of the National Oil and Wheat Flour Fortification Program in Cameroon Using a Program Impact Pathway Approach
Source: Curr Dev Nutr. 2019 Jun 20;3(8):nzz076. doi: 10.1093/cdn/nzz076 (PMC6660062; doi:10.1093/cdn/nzz076)
Supplement: nzz076_Supplemental_Files [file nzz076_supplemental_files.zip › Supplementary_materials.doc]

**Supplementary materials**

Table S1: Wheat flour fortification program impact pathway, definitions, performance criteria and data sources

Table S2: Edible oil fortification program impact pathway, definitions, performance criteria and data sources

Table S3: Number of individual samples added to composite samples of the same brand within each sentinel site
